# Complete genome sequence of *Cytobacillus* sp. strain C11.1 isolated from a soil crust of the Salar de Aguilar high-Andean salt flat, Atacama Region, Chile

**DOI:** 10.1128/mra.00819-26

**Published:** 2026-07-28

**Authors:** Matias Garcia-Mendoza, Javiera Ramos-Zúñiga, Eduardo Lamoza-Galleguillos, Felipe Valenzuela-Ibaceta, Chihhan Hsu-Tsai, Jose Manuel Perez-Donoso

**Affiliations:** 1 BioNanotechnology and Microbiology Laboratory, Center for Bioinformatics and Integrative Biology (CBIB), Facultad de Ciencias de la Vida, Universidad Andres Bello 28087, Santiago, Chile; 2 Pewman Innovation SpA, Santiago, Chile; USDA-ARS San Joaquin Valley Agricultural Sciences Center, Parlier, California, USA

**Keywords:** Salar de Aguilar, high-Andean salt flat, soil crusts, Atacama region, extreme environments, *Cytobacillus*

## Abstract

*Cytobacillus* sp. strain C11.1 was isolated from a microbial soil crust in the Salar de Aguilar, Atacama Desert, Chile. Its complete 4.99-Mb circular genome supports its classification as a *Cytobacillus* species and provides a genomic resource for studying adaptation to hypersaline, high-altitude environments.

## ANNOUNCEMENT

Members of the genus *Cytobacillus* are gram-positive, aerobic, rod-shaped bacteria that inhabit a broad range of terrestrial, marine, and hypersaline environments (1). Several species have been recovered from extreme ecosystems where they are exposed to high salinity, desiccation, ultraviolet radiation, and nutrient limitation (2, 3). Here, we report the whole-genome sequence of *Cytobacillus* sp. strain C11.1 isolated from the Salar de Aguilar, a previously unexplored high-Andean salt flat in northern Chile.

Soil sample C11 was collected from a consolidated evaporitic microbial crust located on the eastern side of Salar de Aguilar (25°48′17.802″S, 68°51′50.616″W), approximately 3,320 m above sea level in the Atacama Region (4, 5). The material was recovered from a depth of 20 cm and consisted of a white botryoidal evaporitic crust with green-pigmented layers. Samples were transported aseptically and stored at −20°C until processing. One gram of material was suspended in 10 mL of autoclave-sterilized distilled water and incubated at 28°C with shaking (160 rpm) for 7 days before plating onto Reasoner’s 2A (R2A) agar containing yeast extract (0.5 g/L), casamino acids (0.5 g/L), peptone (0.5 g/L), glucose (0.5 g/L), sodium pyruvate (0.3 g/L), magnesium sulfate (50 mg/L), and dipotassium phosphate (0.3 g/L). The R2A plates were incubated at 28°C until colonies appeared, and strain C11.1 was subsequently isolated.

Strain C11.1 was grown in Luria-Bertani (LB) broth for 24 h at 28°C. Cells were harvested by centrifugation at 7,000 rpm for 10 min at 4°C, and genomic DNA was extracted using the Wizard Genomic DNA Purification Kit (Promega) according to the manufacturer’s instructions. Whole-genome sequencing was performed using the Oxford Nanopore Technologies MinION platform. Libraries were prepared with the Rapid Sequencing Kit (SQK-RBK004) and sequenced on an R10.4 FLO-MIN114 flow cell using a MinION Mk1B device controlled with MinKNOW v6.8.11. Basecalling was performed with Dorado v7.11.2 (https://github.com/nanoporetech/dorado) in fast mode.

**Fig 1:**
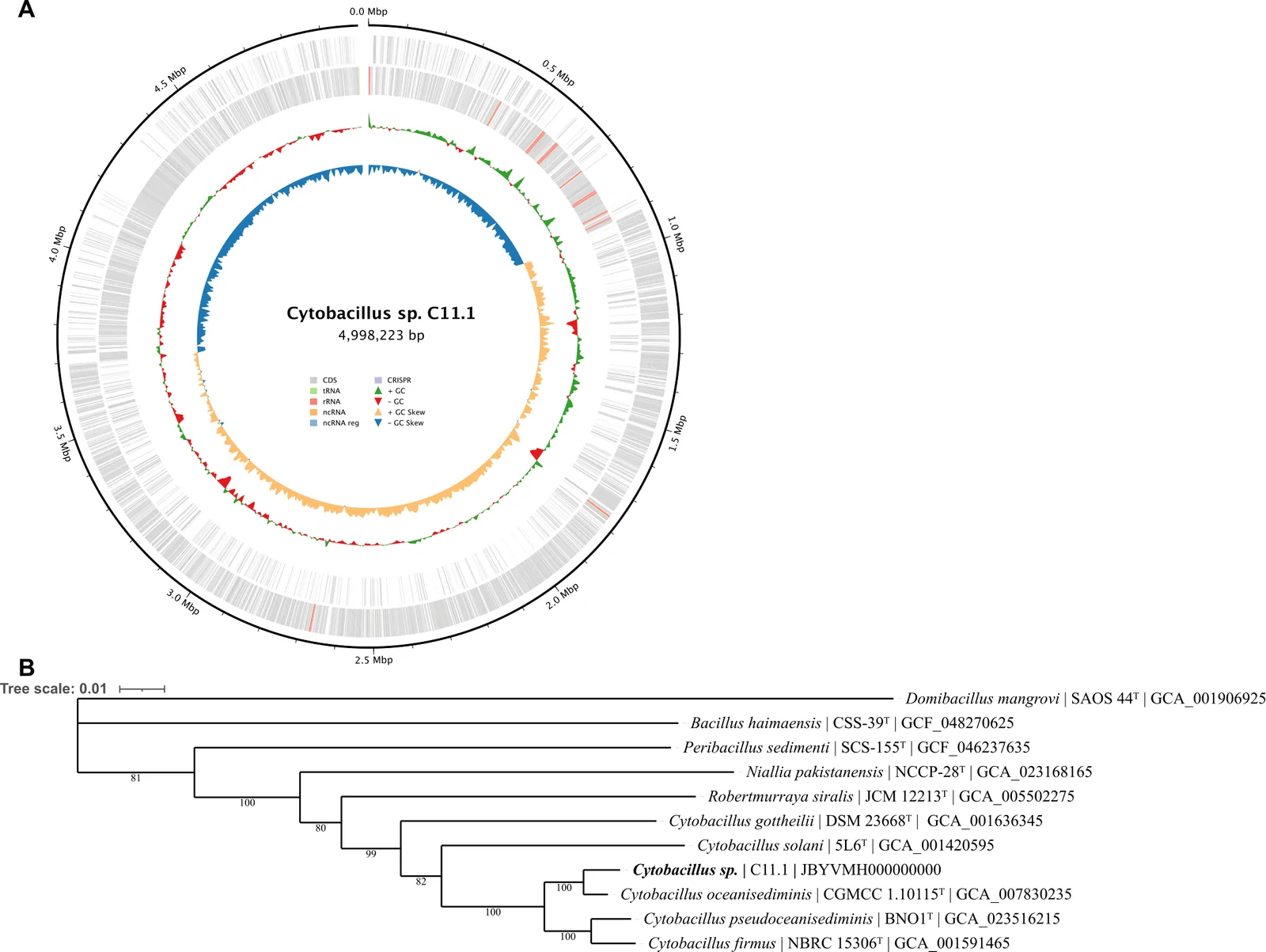
Genomic features and phylogenomic placement of *Cytobacillus* sp. strain C11.1. (**A**) Circular genome map generated with CGView from the Bakta v1.11.0 annotation. From the outside to the center: genomic coordinates (Mbp); predicted coding sequences (CDSs) on the forward and reverse strands (gray); tRNA genes (green), rRNA genes (red), and noncoding RNAs (orange); GC content relative to the genome average (41.2%), with values above and below the average shown in green and red, respectively; and GC skew, with positive values shown in blue and negative values in orange. (**B**) Maximum-likelihood phylogenomic tree inferred from the concatenated alignment of 120 conserved bacterial marker proteins (bac120) identified using GTDB-Tk v2.1.1. The tree was reconstructed with IQ-TREE v2.4.0 under the best-fit substitution model selected by ModelFinder, and branch support was estimated using 1,000 ultrafast bootstrap replicates. Bootstrap values are shown at the nodes, and the scale bar represents substitutions per site.

Raw reads were quality-filtered using NanoFilt v2.8 (6) before *de novo* assembly with Flye v2.9.6 (7), and no additional rotation or reorientation was performed. Assembly quality was evaluated using CheckM2 v1.1.0 (8). Genome annotation was performed using Bakta v1.11.0 (9) (Fig. 1A). Taxonomic placement was assessed using GTDB-Tk v2.1.1 (10), and phylogenomic reconstruction was performed from the bac120 conserved marker set with IQ-TREE v2.4.0 (11) under the best-fit substitution model selected by ModelFinder using 1,000 ultrafast bootstrap replicates rooted at the midpoint (Fig. 1B).

The genome of strain C11.1 consists of a single circular chromosome of 4,998,223 bp with a guanine-cytosine (GC) content of 41.2%. The assembly has 4,928 coding sequences (CDSs)**,** including 4,408 proteins with functional annotation and 408 hypothetical proteins (Table 1). Comparative genome analyses demonstrated that strain C11.1 belongs to the genus *Cytobacillus*. The highest average nucleotide identity (ANI) was 90.13% with *Cytobacillus oceanisediminis* CGMCC 1.10,115ᵀ, whereas digital DNA-DNA hybridization (dDDH) reached 40.5%. Consistent with these genomic metrics, phylogenomic analysis placed strain C11.1 in a strongly supported clade together with *C. oceanisediminis* (bootstrap = 100).

**TABLE 1:** Genome features of C11.1 strain

Parameter	Value
Genome topology	Circular
Genome size (bp)	4,998,223
GC content (%)	41.2
Total raw reads	433,033,423
Completeness (%)	100
Contamination (%)	1.2
Coverage	91×
N50 (bp)	4,998,223
N90 (bp)	4,998,223
Number of contigs	1
*N* ratio (%)	0.0
Coding density (%)	83.9
Coding sequences (CDSs)	4,928
Hypothetical proteins	408
Pseudogenes	78
tRNA genes	109
tmRNA genes	0
rRNA genes	39
ncRNAs	30
ncRNA regions	80
Small ORFs (sORFs)	3
CRISPR arrays	0
Annotation software	Bakta v1.11.0
Annotation database	Bakta DB v6.0

Genome annotation further revealed genes involved in oxidative stress defense, osmotic adaptation, potassium homeostasis, and heavy-metal resistance, including *perR*, *bcp*, glycine betaine transporters (12), *putP*, *trkA/trkH*, and members of the *MerR* family, highlighting the genetic repertoire that may contribute to survival under the hypersaline and high-altitude conditions of the Salar de Aguilar.

## Data Availability

The genome sequence of *Cytobacillus* sp. C11.1 has been deposited in DDBJ/ENA/GenBank under accession GCA_058145115.1 and the raw data under accession SRR39399471 (BioProject PRJNA1474543).
